# Gene Prospector: An evidence gateway for evaluating potential susceptibility genes and interacting risk factors for human diseases

**DOI:** 10.1186/1471-2105-9-528

**Published:** 2008-12-08

**Authors:** Wei Yu, Anja Wulf, Tiebin Liu, Muin J Khoury, Marta Gwinn

**Affiliations:** 1 National Office of Public Health Genomics, Coordinating Center for Health Promotion, Centers for Disease Control and Prevention, Atlanta, GA, USA

## Abstract

**Background:**

Millions of single nucleotide polymorphisms have been identified as a result of the human genome project and the rapid advance of high throughput genotyping technology. Genetic association studies, such as recent genome-wide association studies (GWAS), have provided a springboard for exploring the contribution of inherited genetic variation and gene/environment interactions in relation to disease. Given the capacity of such studies to produce a plethora of information that may then be described in a number of publications, selecting possible disease susceptibility genes and identifying related modifiable risk factors is a major challenge. A Web-based application for finding evidence of such relationships is key to the development of follow-up studies and evidence for translational research.

We developed a Web-based application that selects and prioritizes potential disease-related genes by using a highly curated and updated literature database of genetic association studies. The application, called Gene Prospector, also provides a comprehensive set of links to additional data sources.

**Results:**

We compared Gene Prospector results for the query "Parkinson" with a list of 13 leading candidate genes (Top Results) from a curated, specialty database for genetic associations with Parkinson disease (PDGene). Nine of the thirteen leading candidate genes from PDGene were in the top 10th percentile of the ranked list from Gene Prospector. In fact, Gene Prospector included more published genetic association studies for the 13 leading candidate genes than PDGene did.

**Conclusion:**

Gene Prospector provides an online gateway for searching for evidence about human genes in relation to diseases, other phenotypes, and risk factors, and provides links to published literature and other online data sources. Gene Prospector can be accessed via .

## Background

Variation in the human genome contributes to differences in response to environmental risk factors and disease susceptibility [1]. As a result of the Human Genome Project [2] and advances in new genotyping technology [3], genetic association studies have been flourishing. Recently, genome-wide association studies (GWAS) have begun to systematically examine large numbers of genetic associations [4]. Synthesis of this information is a first step in translating the new knowledge gained from basic research to applications for clinical practice and public health [5].

Although many data sources for genes and diseases are in the public domain, finding published results with potential implications for understanding gene-disease relationships and gene-environment interactions is not a trivial task. Gene Prospector is a Web-based application designed to help researchers prioritize and evaluate evidence for genes related to human disease or interactions with non-genetic risk factors. Gene Prospector provides supporting evidence derived from a curated published literature database [6] and offers quick links to a variety of data sources. Gene Prospector ranks the genes according to the amount of published literature in human genome epidemiology, as well as relevant, published research in two animal (rat and mouse) models. Gene Prospector is a component of HuGE Navigator, an integrated knowledge base for genetics association and human genome epidemiology [7].

## Implementation

### System construction

Gene Prospector was developed as a component of HuGE Navigator, an integrated, searchable, Web-based knowledge base of genetic associations and human genome epidemiology. The HuGE Navigator knowledge base was developed on an open-source infrastructure developed by Yu, et al.[8]. The Gene Prospector was built by using J2EE technology [9] and on other Java open-source frameworks such as Hibernate [10] and Strut [11]. MS SQL server was used as a database server.

### Content extraction and indexing

Published literature in human genome epidemiology is selected from PubMed and deposited in the HuGE Navigator database. The database contains a curated collection of selected PubMed records from 2001 to the present [6]. PubMed records are retrieved from PubMed weekly, such that the database contents on average lag 1 week behind PubMed. Each week, the text mining program developed by Yu, *et al*. [12] is used to perform an initial screen of records newly added to PubMed. The curator then reviews the abstracts and manually indexes each abstract that meets the selection criteria [6] with gene symbols, categories and study types. Once available, MeSH terms for each article are retrieved from the PubMed database using The National Center for Biotechnology Information (NCBI) E-Utilities [13]. The MeSH tree structure [14] is used for efficient record retrieval. To facilitate free text search, the metathesaurus in the Unified Medical Language System is used as a lookup table for term synonyms. Entrez Gene records from NCBI Entrez Gene database [15] are used as standards for gene information. The detailed schema for the literature database can be found in reference [8].

### Gene Selection and Prioritization

The gene list for any search term is generated based on a SQL query of the literature database. For each gene, the numbers of publications in different categories (total, genetic association, genome-wide association, meta-analysis/pooled analysis and genetic testing) are displayed. A ranked gene list is generated by the following heuristic scoring function:

$$\text{Score}=\frac{Hi}{\sum_{i=1}^{n}Hi}+\frac{GAi}{\sum_{i=1}^{n}GAi}+\frac{GWASi}{\sum_{i=1}^{n}GWASi}+\frac{MAi}{\sum_{i=1}^{n}MAi}+\frac{GTi}{\sum_{i=1}^{n}GTi}$$

*Hi*: Number of all publications for a given gene and search term

$\sum_{i=1}^{n}Hi$: Total number of all publications for the search term

*GAi*: Number of genetic association study publications for a given gene and search term

$\sum_{i=1}^{n}GAi$: Total number of genetic association study publications for the search term

*GWASi*: Number of genome-wide association publications for a given gene and search term

$\sum_{i=1}^{n}GWASi$: Total number of genome-wide association publications for the search term.

*MAi*: Number of meta-analysis analysis publications for a given gene and search term

$\sum_{i-1}^{n}MAi$: Total number of meta-analysis analysis publications for the search term

*GTi*: Number of genetic testing publications for a given gene and search term

$\sum_{i=1}^{n}GTi$: Total number of genetic testing publications for the search term

Ranking:

(1). Higher when score is higher;

(2). Higher when animal evidence exists, if score is equal.

### Other Data Sources

For each gene, quick links are provided to key gene-centered databases for general information, published literature, gene variation and expression, pathways, and other data. SNP information for each gene is dynamically retrieved from the dbSNP database and displayed by mutation function categories (nonsynonymous, synonymous, splice site, UTR, intron). Each function category links to detailed information for each SNP. Links to PolyDoms [16] and SNPs3D [17] display prediction analysis for nonsynonymous and synonymous SNPs.

Clicking the PubMed hyperlink dynamically generates a PubMed query combining all relevant gene aliases and protein names. For example, the PubMed query for CCR5 and HIV is generated as follows:

("CCR5" [TIAB] or "CCR5" [mesh term] or "chemokine (C-C motif) receptor 5" [TIAB] or "chemokine (C-C motif) receptor 5" [mesh term] or "CC-CKR-5" [TIAB] or "CCCKR5" [TIAB] or "CD195" [TIAB] or "CKR-5" [TIAB] or "CKR5" [TIAB] or "CMKBR5" [TIAB]) and ((hiv))

Animal study evidence is obtained by querying the Entrez Gene mouse and rat genome databases with the user query. The query term is sent to the NCBI Entrez Gene database via E-Utilities [18]. The returning list of gene symbols from the mouse or rat genome is compared with the given human gene symbol list. The human gene is considered to have animal evidence if the animal gene symbols are found on the human gene list.

### System evaluation and comparison

Parkinson disease was used as a test case because a specialty database is available for comparison. PDGene is a curated, on-line database specific for Parkinson disease that provides updated collections of genetic association studies from the published literature and summaries for each gene related to the disease [19]. PDGene also includes a Top Results gene list; genes are selected for this list based on reported effect size, as described on the PDGene Web site [19]. The Gene Prospector gene list was created by the Gene Prospector query "Parkinson". For an additional comparison, a ranked gene list was generated by the SNPs3D query "Parkinson".

For each of the PDGene Top Results genes, all publications describing genetic associations with Parkinson disease were retrieved from both PDGene and Gene Prospector and the lists were compared.

## Results

### Ascertainment of genetic association studies

Table 1 shows that for the 13 genes on the PDGene Top Results list, we found a total of 299 publications related to Parkinson disease in either PDGene or Gene Prospector. Of these, 140 (46.8%) were shared by both applications. Overall, Gene Prospector captured more of the publications (260 vs 179) because Gene Prospector included types of association studies not included in PDGene, such as genotype-phenotype association studies among affected persons and gene-environment interaction studies.

**Table 1:** Thirteen genes on PDGene top results list: numbers of related publications obtained by querying PDGene and Gene Prospector on 05/25/2008.

**Gene**	**No. Publications** **(Genetic Association Studies)**			
	
	**Total**	**Both**	**PDGene Only**	***Gene Prospector *Only**
	
GBA	14	9	1	4
LRRK2	60	13	2	45
SNCA	51	23	8	20
MAPT	27	19	6	2
PINK1	20	9	2	9
CYP2D6	18	10	2	6
APOE	33	13	2	18
MAOB	21	17	2	2
ELAVL4	4	1	3	0
UCHL1	17	12	4	1
DRD2	19	7	3	8
GSTM1	11	6	1	4
SEMA5A	4	1	3	1
Total	299	140	39	120

Note: Literature published before 2001 was excluded. Literature with publication type "Letter" was excluded. The total number of PubMed publications reporting genetic associations with Parkinson's disease to May 25, 2008 was estimated as 299, the sum of totals in the Common (140), PDGene Only (39) and Gene Prospector Only (120) columns.

### Gene ranking

Nine of the 13 genes on the PDGene Top Results list were found in the top 10th percentile of the Gene Prospector ranked list. In Table 2, we see that 2 of these 13 genes were in the top 10th percentile of the SNPs3D list.

**Table 2:** Thirteen genes on PDGene Top Results list: ranks and rank percentiles generated by querying Gene Prospector and SNPs3D on 06/26/2008.

	**Gene Prospector**		**SNPs3D**	
	
	**Rank Position**	**Rank Percentile**	**Rank Position**	**Rank Percentile**
	
GBA	33	15.3	105	74.5
LRRK2	1	0.4	NA	NA
SNCA	5	2.3	3	2.1
MAPT/STH	2	0.9	28	20.0
PINK1	11	5.1	23	16.3
CYP2D6	7	3.2	NA	NA
APOE	3	1.4	6	4.3
MAOB	13	6.0	25	17.7
ELAVL4	123	57.2	NA	NA
UCHL1	8	2.3	22	15.6
DRD2	25	11.6	104	73.8
GSTM1	43	20.0	133	94.3
SEMA5A	14	6.5	NA	NA

Note: two top genes (GWA 2q36.3 and GWA 7p14.2) were excluded.

NA: Does not exist in the list.

Total number of genes from Gene Prospector: 215

Total number of genes from SNPs3D: 141

### Gene information display and links to integrated evidence

**Figure 1:**
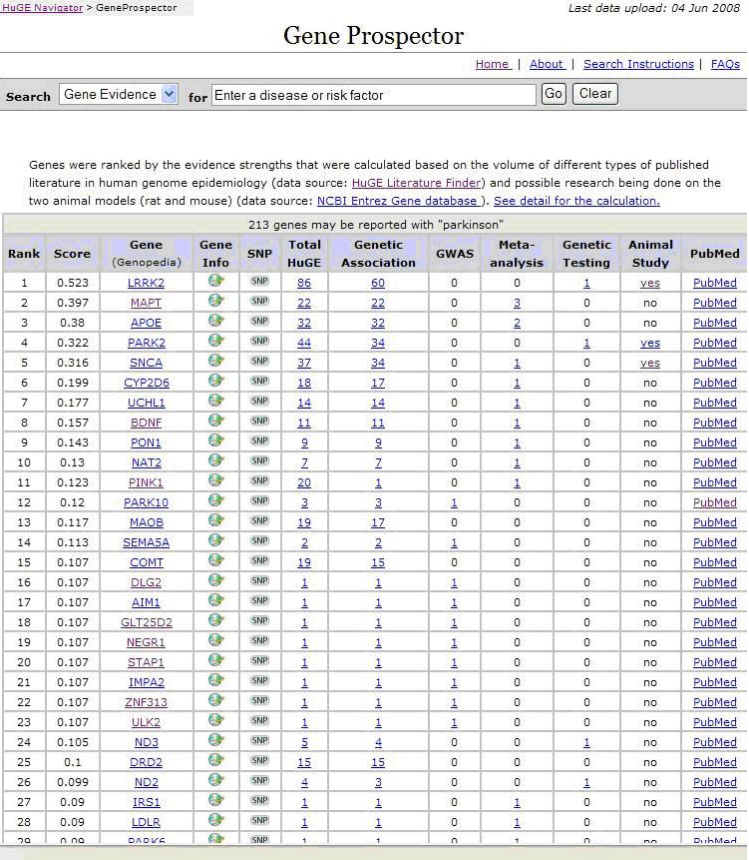
Screen shot of a Gene Prospector search result for Parkinson disease.

Gene Prospector collects and displays relevant information from several major gene-centered databases, as shown in Table 3, and provides quick links to lists of all relevant publications in the HuGE database, as well as to subsets of publications classified as genetic association studies, GWAS, meta-analyses and genetic test evaluations. Gene Prospector also links to SNP information and searches PubMed with a dynamically generated query in Figure 1. As one of the applications in the HuGE Navigator, Gene Prospector easily cross-references other components (e.g., HuGE Literature Finder, Genopedia), further enhancing information retrieval.

**Table 3:** Gene-centered data sources for Gene Prospector.

**Name**	**URL**
**Gene-centered**	

Entrez Gene	
Ensembl Human	
Swiss-Prot	
AceView	
HuGE Navigator	
OMIM	
GeneCards^®^	
Genetics Home Reference	
SOURCE	
PubMed	
**Literature**	
HuGE Navigator	
Genetic Association Database	
**Pharmacogenetics**	
PharmGKB	
**Variation/Prevalence**	
dbSNP	
dbSNP-Genotype	
dbSNP-GeneView	
ALFRED	
SNPper	
Human Gene Mutation Database	
International HapMap Project	
The Cancer Genome Anatomy Project	
**Pathway**	
Kyoto Encyclopedia of Genes and Genomes	
BioCarta	
Pathway Interaction Database	
**Microarray**	
NCBI Gene Expression Omnibus	
**Miscellaneous**	
NCBI Bookshelf	
NCBI Gene Ontology Database	
GeneTests	

## Discussion

Rapid advances in "omic" technologies and basic research have led to discovery of genetic variants, genetic associations, and biomarkers. These advances show promise for translation into applications for clinical practice and health care [5]. Conducting systematic reviews and meta-analyses of population-based genetic association data is an essential approach to synthesizing knowledge for translation. Some recent publications [20,21] have demonstrated the value of this approach; however, this work is usually painstaking and slow. Even now systematic reviews are lacking for many associations [22]. To facilitate such efforts, Gene Prospector has been developed as an evidence gateway to key information sources, selecting genes studied for association with human traits and diseases.

Many gene-centered databases have been developed to gather information related to specific genes. For example, the NCBI Entrez Gene [15] and GeneCard [23] databases attempt to capture all relevant information, including gene-disease associations. However, because they were designed from gene-centered perspective in terms of query functionality, it is not easy to retrieve information related to specific diseases or risk factors. Several different approaches to candidate gene selection have been proposed and implemented. For example, G2D [24] is a bioinformatics tool for predicting genes associated with disease based on multiple information sources, including gene functions in sequence, literature reports, and genetic associations with similar phenotypes. The latter are from a pre-computed list of monogenetic diseases derived from Online Mendelian Inheritance in Man (OMIM) [25], which limits the value of this tool for studies of complex diseases.

SNPs3D is another online database that performs candidate gene selection. SNPs3D applies a heuristic ranking formula to PubMed records downloaded from the NCBI Gene database GeneRIFs (Gene References Into Function) section. In contrast to SNPs3D, Gene Prospector uses a continuously updated and curated data source that is specific for human genetic association studies and classified by publication type, so that more important publications receive greater weight in the scoring formula. Using the PDGene database for comparison, we demonstrated that the Gene Prospector performed better than SNPs3D.

We based our heuristic scoring formula on the total number of publications in the database for a particular gene-disease combination, with additional weight given to four different types of publications: genetic association studies, genome-wide association studies, meta-analyses/pooled analyses, and articles about genetic testing. The added weights reflect the relative importance of such articles in evaluating the evidence for genetic association.

A list of genes ranked by score allows users to see quickly which associations have been studied most often and most systematically. Thus, the main focus of Gene Prospector is not to predict genetic associations with diseases or outcomes but to provide an efficient resource for users seeking to evaluate genetic associations. The Gene Prospector's prioritized gene list for Parkinson overlapped substantially with the Top Results gene list from PDGene, a curated database for genetic association studies of Parkinson disease. Clearly, such a list is no substitute for priorities based on a specialized database curated by a domain expert. However, few such databases currently exist, outside formal research consortia, and even fewer are freely accessible online. However, a prioritized list produced by our scoring strategy may be useful as a starting point for evaluating genetic associations in fields in which specialized resources are not available. As an evidence gateway, Gene Prospector provides a set of links for each candidate gene to curated subsets of published studies (e.g., GWAS); thus, it provides researchers with an information center for quickly and systematically retrieving the evidence needed to evaluate candidate genes for relationships with diseases or risk factors.

The HuGE Navigator database is one of most frequently updated and highly curated literature repositories in the field of genetic association studies. Recently, publications based on GWAS have become a leading source of replicated genetic associations [26]. In collaboration with the Catalog of Published Genome-Wide Association Studies [27], we aim to maintain the most complete and updated collection of GWAS publications. The heuristic scoring function in Gene Prospector gives greater weight to GWAS publications because their abstracts typically feature genes with statistically significant associations. Genes included in meta-analyses also receive extra weight because these labor-intensive analyses tend to be conducted exclusively for associations with the greatest amount of evidence [21].

The Gene Prospector takes advantage of features of the other applications in HuGE Navigator to make information more accessible and easy to navigate; for example, the link to Genopedia provides summaries and quick data links related to the gene. The link to HuGE Literature Finder allows users to continue navigating the information contained in the PubMed abstract of each article. The current version of the Gene Prospector provides information mostly at the gene level, with links to generic information on SNPs. To enhance and enrich the evidence that Gene Prospector can offer, we are in the process of extracting quantitative genetic association data from published meta-analyses, such as numbers of cases and controls, effect sizes, and measures of heterogeneity. The integration of variant-level information into the evidence and scoring system would make Gene Prospector even more useful.

## Conclusion

The Gene Prospector is a unique bioinformatics tool that is seamlessly integrated with other applications in HuGE Navigator. The application provides a central place to obtain information for evaluating genetic associations and conducting translational research. The Gene Prospector presents a wide spectrum of information from molecular biology to published studies, as well as quick links to key genetic data resources.

## Availability and requirements

Gene Prospector is freely available at 

## Competing interests

The authors declare that they have no competing interests.

## Authors' contributions

WY designed and implemented the application, wrote the source codes, and drafted the manuscript. AW participated in design of the system evaluation, data collection and analysis. TL performed data analysis. MJK oversaw the project and revised the draft manuscript. MG provided advice on the project and revised the draft manuscript and led the project. All authors read and approved the final document.
